# Effects of fermentation with different probiotics on the quality, isoflavone content, and flavor of okara beverages

**DOI:** 10.1002/fsn3.3944

**Published:** 2024-01-12

**Authors:** Yixue Li, Hong Song, Zunqin Zhang, Ran Li, Ying Zhang, Lina Yang, Jun Li, Danshi Zhu, Jun Liu, Hansong Yu, He Liu

**Affiliations:** ^1^ College of Food Science and Technology Bohai University Jinzhou China; ^2^ Shandong Yuwang Ecogical Food Industry Co., Ltd. Yucheng China; ^3^ College of Food Science and Engineering Jilin Agricultural University Changchun China

**Keywords:** Okara beverage, probiotics, soybean isoflavones, SPME/GC–MS, storage period

## Abstract

The present study aimed to prepare and evaluate a new probiotic functional beverage, using single‐probiotic and compound probiotic fermentation on okara. Four different forms of fermentation microorganisms used were *Lacticaseibacillus rhamnosus* S24 (Lr), *Lacticaseibacillus paracasei* 6244 (Lp), *Lactobacillus acidophilus* 11,073 (La), and mixed fermentation (Lr + Lp + La). The physicochemical properties, antioxidant activity, flavor change, and storage period of fermented okara beverages with probiotics were investigated. The results showed that different fermentation schemes could significantly improve the physicochemical properties, antioxidant activity, and sensory quality of the okara beverages. The number of viable bacteria in the Lp group (3.53 × 10^8^ CFU/mL), isoflavone content (0.514 μg/mL) were the highest; total phenol and flavonoid content were 3.32 and 5.68 times higher than in the CK group, respectively. DPPH and ABTS^+^ free radical scavenging rates were increased by 11.32% and 20%, respectively (*p* < .05). Through SPME/GC–MS analysis, 44 volatile compounds were identified in the Lr + Lp + La groups, mainly as a result of changes in alcohols and aldehydes produced by fermentation metabolism. It enhances the floral and fruity aroma of the okara beverage. All probiotic‐fermented okara beverages can be stored at 4°C for 15 days, with probiotic activity greater than 10^7^ CFU/mL. This study can obtain a probiotic okara beverage rich in soybean isoflavones and with good flavor. Overall, okara can be used to develop functional beverages containing probiotics and contribute to a zero‐waste approach in the food industry.

## Graphical abstract

This study aims to prove that probiotic fermentation can improve the functional activity of okara beverages, improve taste, and endow fermented okara beverages with different aroma characteristics. With the potential to enrich isoflavone aglycones. In conclusion, the development of probiotic okara beverages contributes to a zero‐waste approach in the food industry.

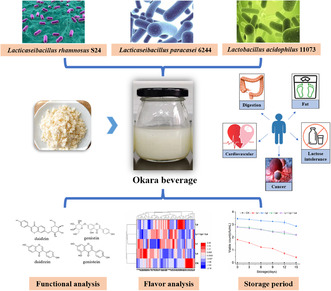

## 1 INTRODUCTION

Okara, a soybean residue, is the main by‐product obtained during soybean processing (Wang et al., 2022). It is usually used as feed for livestock or discarded directly, resulting in a great waste of resources (Li et al., 2018). However, okara is rich in nutrients. Its total dietary fiber content is 50%, protein content is 25%–29%, and fat content is 9%–11% on a dry basis (Crescenti et al., 2013; Jiang et al., 2020). Furthermore, 12%–40% of the soybean isoflavones remain within these by‐products (Nissen et al., 2020). The soybean isoflavones encompass a wide range of benefits, including modulation of host immunity, enhancement of bone mineral density, and alleviation of conditions like acute lung injury and depression (Wang et al., 2023). The combined influence of soybean isoflavones and the gut microbiota actively contributes to the production of various metabolites. In addition, there are studies showing that okara could be used in the food industry to increase the nutritional and functional properties of the product (Ma et al., 2021). At present, many countries have used okara as a source of potentially inexpensive yet functional food ingredients (Kitano et al., 2012; Ni et al., 2019). However, its development was limited due to its high water content, rough taste, and unbearable beany odor.

Probiotics are living microorganisms that benefit the host when consumed in sufficient quantities (Zhang et al., 2019). Studies have shown that probiotic fermentation can improve the flavor and texture of soy products and, concomitantly, enhance their beneficial health properties (Bedani et al., 2015). In addition, probiotic fermentation also affects the content and profile of the isoflavones in the products. For example, fermentation via lactic acid bacteria can significantly increase the content of isoflavone aglycones in soymilk (Wei et al., 2007). Zhu et al. (2019) found that *L. paracasei* had great potential in relation to efficiently enriching bioactive isoflavone aglycones. Compared with unfermented okara, okara products fermented by *Morchella* exhibit higher thermal stability and porous uniformity of structure. The contents of free amino acids, polysaccharides, and total polyphenols have also been significantly improved (Li et al., 2016). At present, most of the probiotic products on the market are carried out using natural or pure milk as the substrate. However, due to the presence of lactose intolerance, dairy allergies, and cholesterol‐rich diets, there is a growing demand for non‐dairy‐alternatives (Kisan et al., 2019). As one of the richest sources of nutrition, soybeans have been reported as highly satisfactory carriers of probiotics (Albuquerque et al., 2017). However, studies on beverages with different probiotics using okara as a fermentation substrate have yet to elucidate its potential.

The aim of this study was to prepare and evaluate a new probiotic functional beverage using okara and probiotics. The effects of different probiotic fermentations on the physical, chemical, and functional properties of okara beverage during the storage period were evaluated. The biotransformation of soybean isoflavones in okara beverages before and after fermentation was analyzed. The distribution of protein and fat particles in okara beverages was observed using confocal laser scanning microscopy. In addition, the effects of different probiotic fermentations on the flavor and organoleptic properties of okara beverages were analyzed by E‐nose and SPME/GC–MS techniques. This research provides theoretical support for the comprehensive utilization of okara and the development of new beverages.

## 2 MATERIALS AND METHODS

### 2.1 Materials

Fresh okaras were provided by the Song Dafang Bean Products Factory (Panjin, China). *Lacticaseibacillus paracasei* 6244 was from the China Center of Industrial Culture Collection (Beijing, China). *Lacticaseibacillus rhamnosus* S24 and *Lactobacillus acidophilus* 11,073 were from Sichuan Gaofuji Biotechnology Co., Ltd. (Sichuan, China). White sugar, anhydrous citric acid, stabilizer, vitamin C, and flavor were purchased from the market. Daidzin, daidzein, genistein, and genistin were from Beijing Soleibao Technology Co., Ltd. (Beijing, China). HPLC‐grade methanol was from Merck (Germany); acetonitrile was from TEDIA (United States) and trifluoroacetic acid was from ACROS (Beijing, China). Methanol and Folin–Ciocalteu were purchased from Sinopharm Chemical Reagent Co. (Shanghai, China).

### 2.2 Microorganisms activation and inoculum preparation

Under aseptic conditions, the three kinds of probiotic lyophilized bacteria powders were placed in MRS broth medium and statically cultured at 37°C for 24 h for activation and rejuvenation. The medium was activated for three generations, and each generation was incubated at 37°C for 24 h. The starter with a viable count of 1 × 10^8^ CFU/mL was obtained. The third‐generation bacterial solution was centrifuged at 4000 r/min for 10 min, and the supernatant was discarded. We washed the precipitated bacterial paste with sterilized 0.85% sterile saline, gave it a good shake, and centrifuged it for 10 min at 4000 r/min; the procedure was repeated three times, and finally, the bacterial paste was mixed with 0.85% sterile saline.

### 2.3 Preparation of the okara beverages

Five different formulations of okara beverages were prepared: a control group was unfermented okara beverage (CK); the four other beverage samples were fermented independently by *Lacticaseibacillus rhamnosus* S24 culture (Lr), *Lacticaseibacillus paracasei* 6244 culture (Lp), *Lactobacillus acidophilus* 11,073 culture (La), and a microbial mix containing equal proportions of the aforementioned strains (Lr + Lp + La). The preparation method used to create the fermented okara beverage was as previously published, with minor modifications (Voss et al., 2021). Fresh okaras were deodorized with 4% edible alkali, heated in a water bath at 90°C for 30 min, and the excess alkali was neutralized with a citric acid solution. The ratio of okara and water was 1:10 (v/v), and it was passed through a wet pulverizer to make okara puree. Stabilizers and excipients were added, and the mixture was then homogenized (Shanghai Specimen model Factory, China) for 20 min, and autoclaved at 121°C for 20 min. After cooling, probiotic starter with a 3% viable bacteria count of 1 × 10^8^ CFU/mL was inoculated on a standard clean table (Sujing, Antai) and incubated at 37°C for 24 h.

### 2.4 Physical and chemical analysis

#### 2.4.1 Determination of solid content

A handheld refractometer was used to determine the solid content of the probiotic bean residue beverage.

#### 2.4.2 Determination of dietary fiber content

The dietary fiber content was measured according to the previous research method, with some modifications (Yang et al., 2014).

#### 2.4.3 Soluble protein and free amino acid analysis

The soluble protein content of the okara beverages was determined using the Coomassie brilliant blue method (Sheih et al., 2014), with BSA (bovine serum albumin) used as the standard. The soluble protein content in the samples was expressed in BSA equivalents (mg/mL). Free amino acids were detected based on previous research methods with minor modifications (Liu et al., 2018), using glycine as the standard. Absorbance was measured at 570 nm using a UV‐2500 spectrophotometer (Shimadzu, Tokyo, Japan).

#### 2.4.4 Ultra‐high‐performance liquid tandem mass spectrometer (UPLC‐MS/MS) analysis

According to the UPLC‐MS/MS method (Fernandes et al., 2017; Huang et al., 2021; Lee et al., 2018), the content of the soybean isoflavones in each sample was determined, and some adjustments were made to this technique. Fifteen milliliter of each sample were placed into conical flasks, 35 mL of 80% methanol (w/v) was added, and the mixture was sonicated for 40 min. The extract was then centrifuged at 5000 *g* (Anting Co., Shanghai, China) for 12 min at 4°C and concentrated via rotary evaporation. One milliliter of each sample was taken, diluted with a 10% (w/v) methanol solution, and centrifuged at 12000 rpm for 10 min at 4°C. After centrifugation, the supernatant was taken and passed through a 0.22 μm filter membrane for UPLC‐MS/MS analysis.

Column parameters: ACQUITY UPLC BEH C18, 1.7 μm, 2.1*100 mm; A solution: ddH_2_O; B solution: acetonitrile; flow rate: 0.3 mL/min; injection volume: 5 μL; column oven: 40°C; data were collected in positive ion mode.

Calibration curves were performed using genistein, daidzein, genistin, and daidzin. A repeatability test was carried out for each sample.

#### 2.4.5 Total phenolic and flavonoid content determination

The total polyphenols present in the okara beverage samples were measured using the Folin–Ciocatleu reagent, and absorbance was measured at 750 nm using a UV‐2500 spectrophotometer (Shimadzu, Tokyo, Japan). The results were expressed as mg gallic acid equivalent (GAE) per mL of okara beverage. The colorimetric determination of total flavonoids was measured as previously described, with minor modifications (Alide et al., 2020), with a UV‐2500 spectrophotometer (Shimadzu, Tokyo, Japan), where absorbance was measured against a blank at 510 nm. The flavonoid content in the samples was expressed in rutin equivalents (mg/mL).

### 2.5 Antioxidant activity

The determination of the DPPH radical scavenging activity of the okara beverages was measured as previously described (He et al., 2020). Results were expressed as moL Trolox equivalents per liter of okara beverage samples. The absorbance was measured at 517 nm using a UV‐2500 spectrophotometer (Shimadzu, Tokyo, Japan).

The scavenging activity of okara beverages was determined using the ABTS^+^ radical method (Xiao et al., 2015). The absorbance was measured at a wavelength of 734 nm using a UV‐2500 spectrophotometer (Shimadzu, Tokyo, Japan).

### 2.6 Flavor analysis

#### 2.6.1 E‐nose analysis

The E‐nose analysis of odors was performed according to the method of Yang et al., (2016), with minor modifications.

#### 2.6.2 SPME‐GC–MS analysis

The analytical method was as described previously (Dongmo et al., 2017; Teerawat et al., 2023), with minor modifications. Five milliliter of the sample was added to a 15 mL headspace flask with a teflon diaphragm and treated in a water bath at 55°C for 10 min. A needle with a manual SPME injection handle was inserted into the flask. After 30 min, it was inserted into the GC–MS injector and resolved at 250°C for 5 min.

Volatile compounds were analyzed using GC–MS apparatus (7890A/5975C, Agilent Technologies, Santa Clara, CA, USA). A HP‐5MS elastic quartz capillary column (30 m × 0.25 mm × 0.25 μm) was used with an inlet temperature of 250°C and a flow rate of 1.0 mL/min without split injection. The programmed heating method was as follows: the initial temperature of the cylinder is kept at 35°C for 5 min, then increased to 65°C at 2°C/min, then increased to 110°C at 3°C/min, and finally increased to 230°C at 10°C/min for 5 min. The electron energy was 70 eV, the ion source temperature was 200°C, the emission current was 200 μA, the transmission temperature was 250°C, the detection pressure was 350 V, and the data were collected as a full scan.

#### 2.6.3 Sensory evaluation

As previously described, 5 okara beverages were sensory assessed by 20 participants from Bohai University, including students and faculty employees, who were trained in how to do sensory assessments (10 males and 10 females) (Kim et al., 2020). Pre‐test sessions were carried out to define the list of descriptors to be evaluated and the intensity range, and to verify the reliability, consistency, and discriminating ability of panelists when testing the products. The beverages were assessed using a 6‐point hedonic scale for taste, aroma, texture, appearance, and overall acceptability. The rating ranged from very dislike (1 point) to very like (6 points). The differing probiotic‐fermented or non‐fermented okara beverages were consumed at 4°C in a clear glass. Participants rinsed their mouths with mineral water between samples.

### 2.7 Rheological analysis

The apparent viscosity and frequency sweep were determined using a rheometer (Discovery HR‐1, TA, USA) equipped with an aluminum plate (40 mm diameter). This technique was based on previous research (Jiao et al., 2018), with minor modifications. The temperature used was 4°C, and the shear rate was in the range of 0.1–1000 rad/s to analyze the change of apparent viscosity with shear rate. Variation of storage modulus (*G'*) and loss modulus (*G"*) was over a frequency sweep range of 1–100 Hz.

### 2.8 Microstructure

The distribution of fat and proteins in samples was observed using a confocal laser scanning microscope (Ningtyas et al., 2021). The protein was stained with Nile blue (0.05% w/w in ethanol) dye and excited with a 633 nm laser. The fat was stained with Nile red (0.1% w/w in acetone) and excited under a 515–530 nm laser to double‐stain the sample. Ten microliter of each staining solution was added into a 100 μL sample, mixed thoroughly with a vortex mixer, stained the sample for 5 min, then loaded the 10 μL stained sample onto a 26 × 76 mm slide, and covered it with an 18 × 18 mm cover slip. A 40 × magnifying lens was used to observe fat and protein molecules.

### 2.9 Evaluation of the storage period of fermented okara beverages

After processing (autoclave or fermentation), the okara beverages were placed in sterile 100 mL bottles and stored in cold storage (4°C) for 15 days. Samples were taken from these stocks every 3 days to explore any changes in pH value, titratable acidity, total sugar, dehydration rate, and viable bacteria content. The non‐fermented okara beverage was used as a control.

#### 2.9.1 Physicochemical analysis

Physicochemical properties were determined for each sample during storage, including pH, titratable acidity, total sugar, and dehydration rate. Potentiometric determination of pH was performed using a pH meter (Shanghai Yi Electrical Scientific Instrument Co., Ltd., China).

Titratable acidity (g of acid/100 mL) was determined by titration of a 5 mL aliquot with a 0.01 mol/L NaOH solution using 1% phenolphthalein as an indicator.

The phenol–sulfuric acid method was utilized to determine the total sugar content of the okara beverages. Combined with the actual situation, the dilution ratio of the sample extract was improved.

5 mL of samples were centrifuged at 5000 r/min for 15 min. The supernatant was discarded, and the sediment was accurately weighed. The following formula was used to calculate the dehydration rate.

$$\text{dehydration rate}\;\left(\%\right)=\frac{m_{1}}{\;m_{2}}\times 100\%$$

where *m*_1_ and *m*_2_ represent the mass of the precipitate after sample centrifugation and the 5 mL sample mass, respectively.

#### 2.9.2 Microbiological analysis

The okara beverage was sampled for gradient dilution, and the number of viable bacteria was determined by pouring culture into MRS medium. It was then incubated in an incubator at 37°C for 48 h, using 3 parallel plates per sample. Consider plates with counts ranging from 30 to 300 CFU/mL. Viability tests were analyzed by plate counting (Al‐Sahlany et al., 2023; Kareem et al., 2017; Niamah et al., 2023).

### 2.10 Statistical analysis

Origin 2018 and IBM SPSS 22.0 were used for image rendering and data processing. The differences between the mean values were tested by the least significant difference (LSD) method of one‐way analysis of variance (ANOVA), and *p* < .05 was considered to be statistically significant. All tests were repeated three times (*n* = 3), and values were expressed as mean ± standard deviation.

## 3 RESULTS AND DISCUSSION

### 3.1 Physicochemical properties observed in fermented beverages

As shown in Table 1, the increase in the content of soluble dietary fiber (SDF) in the fermented okara beverage may be due to the breakage of the cellulose glucosidic bond and the formation of a new reducing end, which degrades part of the insoluble dietary fiber (IDF) into soluble dietary fiber. Solid matter content mainly showed a continuous decreasing trend, which was related to the change in microbial count. The content of free amino acids in Lr + Lp + La (7.94 ± 0.28 mg/mL) and Lp (7.48 ± 0.15 mg/mL) groups was significantly higher than that in the CK group (3.20 ± 0.08 mg/mL) (*p* < .05). In contrast, in the single fermentation group, the total free amino acids in the Lp group increased by 4.28 mg/mL, indicating that *Lacticaseibacillus paracasei* 6244 had strong proteolytic ability. The content of soluble protein in the CK group was 1.12 ± 0.06 mg/mL. Compared with the CK group, the soluble protein content of probiotic‐fermented okara beverage was significantly increased (*p* < .05), which was due to the continuous action of carbohydrates on the cell wall of okara and the microbial decomposition of okara proteins and peptides (Xiao et al., 2021). Among them, the Lr + Lp + La groups have the strongest protein transformation abilities.

**TABLE 1:** Determination results of the physical and chemical properties of okara beverages.

Sample	Solid matter (g/100 g)	Soluble dietary fiber (%)	Free amino acid (mg/mL)	Soluble protein (mg/mL)
CK	20.07 ± 0.74^a^	1.17 ± 0.35^b^	3.20 ± 0.08^e^	1.12 ± 0.06^d^
Lr	19.13 ± 0.57^ab^	2.10 ± 0.36^a^	6.49 ± 0.07^d^	1.60 ± 0.06^c^
Lp	18.97 ± 0.57^b^	2.73 ± 0.45^a^	7.48 ± 0.14^b^	2.03 ± 0.12^b^
La	18.67 ± 0.47^b^	2.53 ± 0.49^a^	7.15 ± 0.11^c^	1.89 ± 0.07^b^
Lr + Lp + La	18.47 ± 0.31^b^	2.80 ± 0.36^a^	7.94 ± 0.28^a^	2.43 ± 0.05^a^

*Note*: Different lowercase letters in the same column a–e indicate significant difference (*p* < .05).

### 3.2 The change in soybean isoflavone contents in different samples before and after fermentation

**FIGURE 1:**
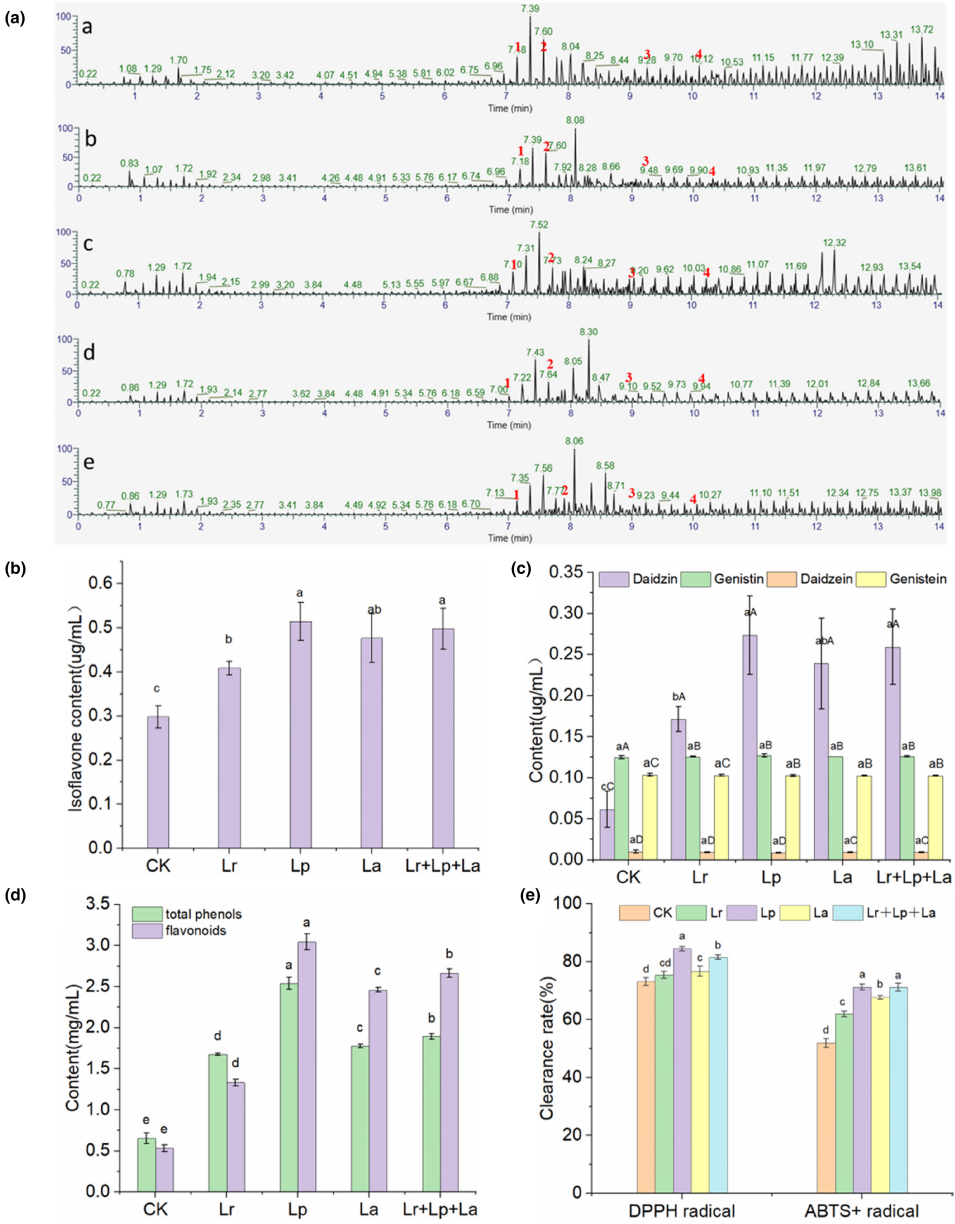
Effect of probiotic fermentation on the functional activity of okara beverages. (a) UPLC‐MS/MS relative abundance; (b) total soybean isoflavone content; (c) individual isoflavone fractions; (d) total phenolic flavone content; (e) antioxidant activity. a. CK; b. Lr; c. Lp; d. La; e. Lr + Lp + La. 1. Daidzin (7.08 min). 2. Genistin (7.80 min). 3. Daidzein (9.09 min). 4. Genistein (10.17 min). Different lowercase letters in the same column a–e indicate a significant difference (*p* < .05). Different capital letters in the same row A–D indicate a significant difference (*p* < .05).

Four soybean isoflavone components, daidzin, genistin, daidzein, and genistein, were detected in the okara beverage extracts using UPLC‐MS/MS analysis. Quantified by the relative abundance chromatogram of okara beverage, the characteristics and retention time of each peak are shown in Figure 1a.

The total isoflavone content of the okara beverages after probiotic fermentation was 0.409, 0.514, 0.476, and 0.498 μg/mL; these were significantly higher than that of the CK group (0.298 μg/mL) (Figure 1b). Studies have shown that the biotransformation capacity of isoflavones is related to the survival rate of bioactive strains in fermentation samples (Champagne et al., 2010). As shown in Figure 1c, before fermentation, the CK group showed 0.06 μg/mL of daidzin, 0.13 μg/mL of genistin, with a small amount of daidzein and genistein of 0.01 and 0.10 μg/mL respectively. The contents of daidzin and genistin in the CK group and Lr + Lp + La group were 0.27 μg/mL, 0.13 μg/mL and 0.26 μg/mL, 0.13 μg/mL, respectively. After fermentation with probiotics, the content of glycoside isoflavones was gradually decreased, whereas the content of aglycone isoflavones was steadily increased. The decreasing trend of glycoside isoflavones was greatest after 24 h of fermentation, which may be closely related to the increase of β‐glucosidase activity.

Fermentation significantly increased the content of isoflavone aglycones; the biological transformation effect of mixed‐strain fermentation was better than that of single‐strain fermentation. The selection of bacteria is the key to fermented okara beverages. In single‐strain fermentation, the content of aglycones in the Lp group was the highest (0.401 μg/mL) (Figure 1c), which was significantly higher than other strains. The level of aglycones after fermentation of the Lp group was twice that of the CK group. *Lacticaseibacillus paracasei* 6244 had an advantage in okara beverages.

### 3.3 Analysis of total phenolic and flavonoid and their antioxidant activities

As can be seen in Figure 1d, the content of total phenols and flavonoids in the okara beverages fermented with the different probiotic combinations was different. The contents of total phenolics and flavonoids within the okara beverage fermented by the probiotics were significantly higher than those in the CK group (0.71 ± 0.01 mg/mL and 0.53 ± 0.04 mg/mL) (*p* < .05). The contents of total phenols and flavonoids in the Lp group were 2.54 ± 0.08 mg/mL and 3.04 ± 0.10 mg/mL, respectively, while those in the Lr + Lp + La groups were 1.89 ± 0.35 mg/mL and 2.66 ± 0.05 mg/mL, respectively. The content of phenolic substances in the Lr + Lp + La group increased, which may be due to the synergistic effect of probiotic compound fermentation, which increased the enzymes produced by metabolism and promoted the release of phenolic and flavonoid. This was consistent with the results of Lai et al. (2013).

The antioxidant activities of different probiotic‐fermented okara beverages were evaluated by DPPH radical and ABTS^+^ radical scavenging assays. Compared with the CK group, the ability of the probiotics to scavenge DPPH radical and ABTS^+^ radical after fermentation was significantly improved (Figure 1e). It may be attributed to the increase in total phenolic and flavonoid (Figure 1d). Relevant studies have shown that probiotic fermentation significantly increases the total phenolic content, and there is a certain linear relationship between the antioxidant capacity and total phenolic content during the fermentation process (Chen et al., 2021). Based on the indicators of antioxidant capacity in the four groups of samples, the Lp group had the strongest antioxidant capacity. The scavenging rates of DPPH radical and ABTS^+^ radical were 84.45% and 71.19%, respectively.

### 3.4 Effects of probiotic fermentation on the sensory properties of okara beverages

#### 3.4.1 E‐nose analysis

**FIGURE 2:**
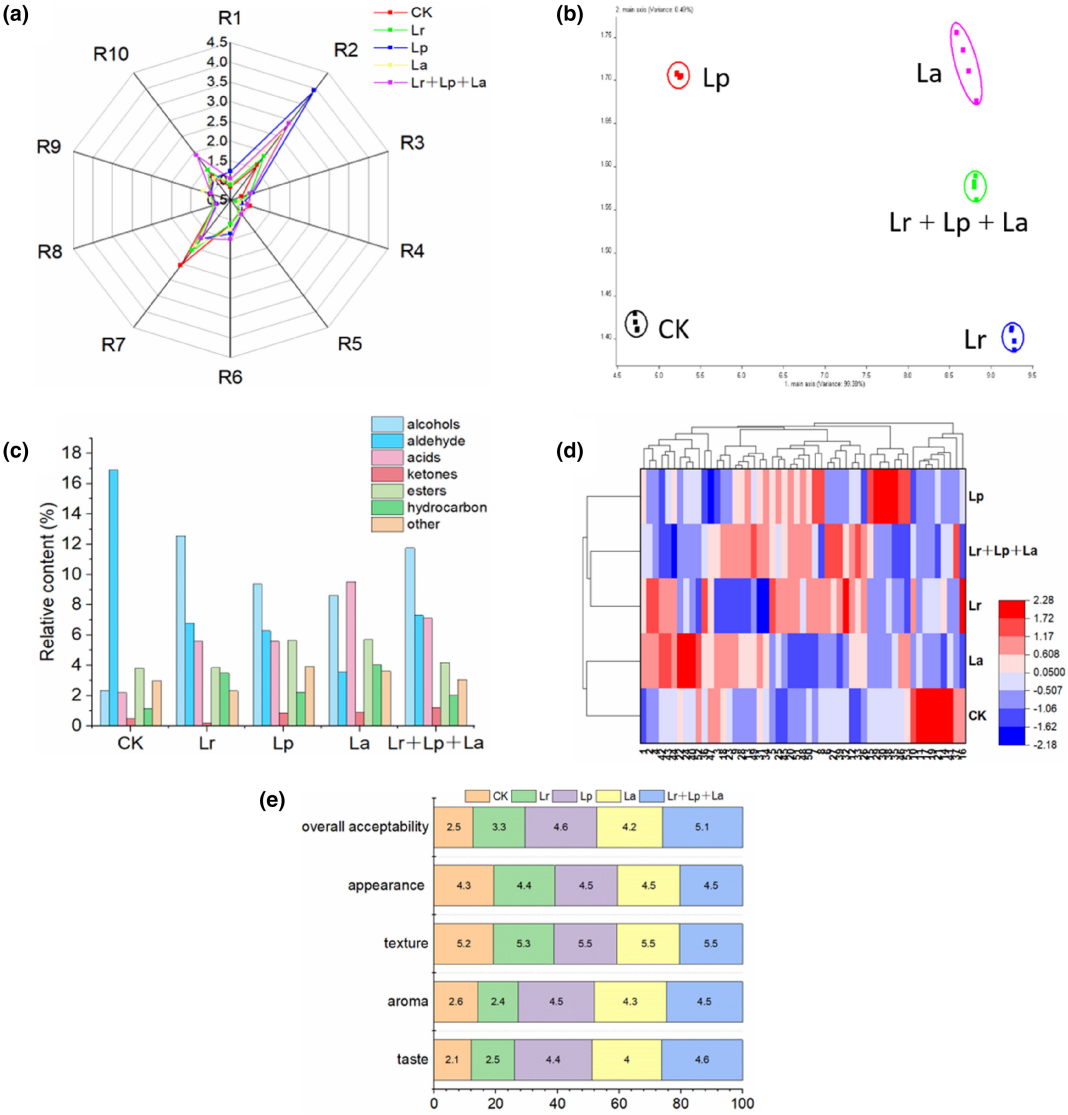
Effect of probiotic fermentation on the flavor of okara beverages. (a) E‐nose radar chart; (b) principal component analysis chart; (c) relative content of volatile compounds; (d) clustering heat map; (e) sensory analysis. R1–R10 represent the response characteristics of each sensor. R1: sensitivity to aromatic compounds; R2: sensitive to nitrogen oxides; R3: sensitive to ammonia and aromatic compounds; R4: sensitive to hydrogen; R5: sensitive to alkenes and aromatic compounds; R6: sensitive to hydrocarbons; R7: sensitive to hydrogen sulfide; R8: sensitive to alcohols and some aromatic compounds; R9: sensitive to aromatic compounds and organic sulfides; R10: sensitive to alkanes (methane, etc.).

Radar fingerprint chart of the volatile compounds in okara beverages at different probiotic fermentations is shown in Figure 2a. A significant difference was observed between the Lp group and the CK group, which suggested the noticeable alteration of volatile compounds in okara beverages during the fermentation process. R2 (nitrogen and oxygen compounds) was most closely related to the odor change in okara beverages fermented by different probiotics. Fermentation with probiotics significantly increased its content; the content of R7 (sulfide) was significantly decreased (*p* < .05), while the content of other compounds had no significant change. R2 and R7 sensor signals in the Lp and Lr + Lp + La groups showed significant differences (*p* < .05). The content of sulfide in food is very small, and the taste threshold is low, but it contributes a lot to processed cereals. It is mainly formed through the Maillard reaction in the processing process. Therefore, the addition of starter culture can inhibit the Maillard reaction to a certain extent.

The results were statistically analyzed using PCA to highlight the differences in the volatile profiles. It demonstrated that the total contribution rate above 85% indicated the good feasibility of the method (Yang et al., 2016). The E‐nose principal component analysis diagram (PCA) of 5 groups of samples is shown in Figure 2b. The contribution rates of the first principal component and the second principal component (PC1, PC2) were 99.38% and 0.49%, respectively. The accumulative variance contribution rate of the first two PCs was 99.87% (more than 85%), which indicated that the two principal components included most information about volatiles and compounds. There was no overlap between the different samples. Except for R3, R4, R5, and R8, the other sensor signals showed significant differences between the samples (*p* < .05), indicating that fermentation caused significant changes in volatile flavor substances. These results suggest that PCA of E‐nose data can be used to distinguish volatile components of fermented okara beverages with different probiotics.

#### 3.4.2 Volatile components measured by SPME/GC–MS

In order to evaluate the effect of fermentation with different probiotics on the flavor of Okara beverage, the volatile compounds of 5 samples were analyzed by SPME/GC–MS. The total relative contents and cluster analysis results of different classes of volatile compounds in all samples are shown in Figure 2c,d. A total of 53 volatile compounds were detected in 5 samples. Volatile compounds were divided into 7 groups according to their chemical properties, including 9 alcohols, 12 aldehydes, 9 acids, 4 ketones, 6 esters, 7 alkanes, and others. The detailed volatile groups of their relative contents are listed in Table 2. There were 30 kinds of volatile flavor substances in the CK group, 37 kinds in the Lr group, 38 kinds in the Lp group, 36 kinds in the La group, and 44 kinds in the Lr + Lp + La group. 2‐Hexenal (E)‐ and 2,4‐decanodienal (E,E)‐ were found to be the predominant components in the CK group. The relative content of 2,4‐decanodienal (E,E)‐ was 10.55%, which has an unpleasant taste. Due to its low odor threshold, it exhibited great influence on the flavor and was the main odor component in the okara beverage. After fermentation, the content of 2,4‐decanodienal (E,E)‐ in the Lp group was 0.67%, while no 2,4‐decanodienal (E,E)‐ content was detected in the Lr + Lp + La group. In the Lp group, 1‐hexanol, methyl caprylate, 2‐propylene, hexanoic acid, etc. were the main skeleton of the aroma components, and most of the bitter and fishy ingredients were consumed during the fermentation process, which greatly improved the flavor of okara beverages. After the synergistic fermentation of the three strains, there was synergistic promotion of the metabolism of the strains, which enhanced the metabolic activity. The change in aroma components was the reason for the different flavors of probiotic single bacteria and synergistic fermentation.

**TABLE 2:** Volatile compounds in fermented okara beverages with different probiotics identified by SPME/GC–MS.

Number	Volatile compounds	Relative content (%)				
		CK	Lr	Lp	La	Lr + Lp + La
1	1‐Hexanol	0.76	3.94	3.55	4.84	3.70
2	1‐Octanol	0.91	1.63	0.99	1.48	1.24
3	1‐Octen‐3‐ol	0.48	ND	ND	0.71	0.56
4	1‐Heptanol	0.21	0.46	0.28	0.46	0.32
5	1‐Nonanol	ND	3.56	2.31	1.00	2.51
6	2,4‐Decadien‐1‐ol	ND	1.34	ND	ND	2.17
7	trans‐2‐Undecen‐1‐ol	ND	1.59	2.09	ND	1.09
8	3‐Nonen‐1‐ol, (Z)‐	ND	0.04	0.07	ND	ND
9	L. alpha.‐Terpineol	ND	ND	0.10	0.13	0.15
10	Octanal	0.85	0.66	ND	0.27	0.52
11	Heptanal	0.35	0.11	0.06	0.13	0.14
12	2‐Heptenal, (Z)‐	ND	0.71	0.43	0.58	0.89
13	Furfural	0.60	0.72	1.33	1.20	1.48
14	2‐Hexenal, (E)‐	0.13	0.06	0.03	ND	0.06
15	2‐Nonenal, (E)‐	ND	ND	1.13	ND	0.59
16	Decanal	0.24	0.38	0.09	ND	ND
17	Nonanal	1.74	0.77	0.33	0.37	0.60
18	5‐Ethyl‐2‐furaldehyde	0.05	ND	ND	0.11	0.11
19	2,4‐Nonadienal, (E,E)‐	2.41	0.43	ND	ND	0.47
20	2,4‐Decadienal, (E,E)‐	ND	1.96	2.21	ND	2.46
21	2,4‐Decanodienal, (E,E)‐	10.55	0.99	0.67	0.90	ND
22	Hexanoic acid	1.47	1.20	0.85	3.10	1.49
23	Octanoic acid	0.72	0.60	0.90	2.31	2.31
24	Nonanoic acid	ND	0.66	0.32	2.22	0.33
25	Hexanoic acid, 2‐propenyl ester	ND	2.90	3.17	1.60	2.39
26	Acetic acid	ND	0.13	ND	0.12	0.16
27	2‐Octenoic acid	ND	0.08	ND	ND	0.21
28	2‐Octenoic acid, (E)‐	ND	ND	0.17	0.18	0.25
29	2‐Tridecenoic acid, (E)‐	ND	ND	0.13	ND	ND
30	Trans‐2‐heptenoic acid	ND	ND	0.06	ND	ND
31	2‐Nonanone	0.24	ND	0.30	0.38	0.44
32	2‐Tridecanone	ND	0.17	ND	ND	0.10
33	Benzophenone	ND	0.03	0.02	0.03	0.05
34	2(3H)‐Furanone, dihydro‐5‐pentyl‐	0.25	ND	0.54	0.47	0.64
35	1,2‐Benzenedicarboxylic acid, butyl 2‐methylpropyl ester	ND	ND	0.07	0.02	ND
36	Hexadecanoic acid, methyl ester	0.01	0.03	ND	0.02	0.02
37	Hexanoic acid, hexyl ester	0.12	ND	ND	ND	0.22
38	Octanoic acid, methyl ester	3.66	3.79	5.58	3.36	3.92
39	Dibutyl phthalate	0.01	0.03	ND	ND	0.04
40	Allyl heptanoate	ND	ND	ND	2.29	ND
41	Cyclotrisiloxane, hexamethyl‐	0.13	ND	ND	0.05	0.05
42	Cyclohexasiloxane, dodecamethyl‐	0.35	0.97	ND	1.17	0.24
43	Cyclopentasiloxane, decamethyl‐	0.46	1.42	0.69	2.06	0.38
44	Cycloheptasiloxane, tetradecamethyl‐	0.18	0.51	0.34	0.54	ND
45	Cyclononasiloxane, octadecamethyl‐	0.03	0.09	0.08	0.06	0.09
46	Tetradecane	ND	ND	0.08	0.05	ND
47	Eicosane	0.01	0.01	ND	0.01	0.01
48	1,3‐Hexadiene, 3‐ethyl‐2‐methyl‐	ND	0.49	0.99	ND	1.08
49	Phenol, 2,4‐bis(1,1‐dimethylethyl)‐	ND	ND	0.05	0.09	0.17
50	Anethole	0.13	0.46	0.29	ND	0.55
51	Benzyl nitrile	ND	0.21	0.18	ND	0.25
52	Oxime‐, methoxy‐phenyl‐	0.49	ND	0.30	0.80	0.22
53	Furan, 2‐pentyl‐	2.35	1.64	3.11	2.75	1.88

Abbreviation: ND, not detected.

In this study, the total amount of alcohol in the fermented samples was at least 4 times that of the unfermented beverages (Figure 2c), indicating that most of the alcohols were produced by probiotic fermentation. Compared with the unfermented samples, 1‐hexanol (fruit aroma) was significantly increased, especially in the La group (*p* < 0.05). The result was similar to the previous study: probiotic fermentation significantly increased the content of alcohols in elderberry juice (Ricci et al., 2018). Compared with the CK group, the relative contents of (E)‐2‐hexenal and (E, E)‐2,4‐decanodienal were decreased after fermentation (*p* < .05). This result shows that aldehydes produced by probiotics can bring positive aroma properties to the flavor of beverages. The acids were mainly derived from fermentation by‐products. A total of 9 acids were detected in the fermentation samples, including 7 acids in Lp and Lr + Lp + La groups, and only 2 acids in CK. The total relative content of acids ranged from 2.19% to 9.53%, with the highest content in La (Figure 2c). Among the ketones we detected, 2‐tridecanones and benzophenones were produced only after fermentation. Ester compounds were formed by esterification of short‐chain free acids and alcohols, which have a fruity aroma and sweet flavor. These esters have a positive effect on the aroma of okara beverages. A total of 6 esters were identified in this study, and the slight increase in ester levels found in fermented beverages may be related to the increased availability of alcohol precursors (Chen et al., 2017). The compound that most significantly affected the flavor of fermented beverages was methyl octanoate (Table 2). The Lp group had the highest relative content (5.58%). In addition, 2‐pentylfuran has pleasant aroma properties associated with fruits and vegetables. Probiotic fermentation retains or strengthens some of the inherently volatile compounds in okara beverages, such as 1‐hexanol and furfural, while new flavor compounds such as nonanoic acid and some ketones are produced through the fermentation process.

A hierarchical cluster was used as a preliminary method to assess the quantitative relationship among the aromatic profiles of the different samples based on their Euclidean distance (Figure 2d). The samples were characterized, and the results showed that probiotic fermentation had a significant effect on the flavor characteristics of okara beverages. The Lp group had a more significant effect on the volatile characteristics of okara beverage than other strains.

#### 3.4.3 Sensory evaluation

The okara beverages were assessed using a 6‐point hedonic scale for taste, aroma, texture, appearance, and overall acceptability. The sensory scores are shown in Figure 2e. The taste, aroma, and overall acceptability of the Lr + Lp + La group were significantly higher than those of the CK group, followed by the Lp group (*p* < .05). In addition, there was no significant difference in appearance and texture between fermented okara beverages. The GC–MS aroma characteristics of Lp and Lr + Lp + La groups were different from other beverages, with more variety and relative content of aroma volatile compounds, resulting in higher sensory scores for flavor, and lower sensory scores for the Lr group, which was also consistent with the physicochemical test. The color, taste, aroma, and sensory quality of fermented soybean residue drinks with probiotics were improved. Due to the different metabolites produced by the probiotics in the fermentation process, the types and contents of enzymes and compounds produced by different strains in the metabolic process are different, and hence the taste of substances of different samples are different (Chen et al., 2019). At the same time, due to the synergistic effect between different probiotics, the nutritional content of fermented beverages can be improved and these changes will directly affect the sensory quality of beverages. The sensory score of mixed fermentation was higher than that of single fermentation. It was worth noting that the peculiar smell of the fermented okara beverages, such as beany flavor, was significantly improved, and provided the characteristic smell of the fermented okara beverages. Similarly, the odor of fermented bean curd whey can be significantly improved after fermentation by probiotics, and the characteristic odor of fermented bean curd whey is provided (Zhu et al., 2019).

### 3.5 Rheological analysis

**FIGURE 3:**
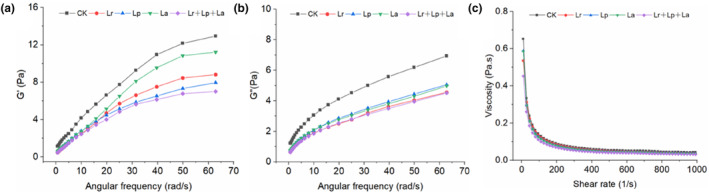
Effect of probiotic fermentation on the rheological properties of okara beverages. (a) Storage modulus G′; (b) Loss modulus G″; (c) Apparent viscosity.

The degree of bond recombination was observed by measuring the elastic modulus, loss tangent, and yield stress of probiotic okara beverages so as to determine their stability. It can be seen from Figure 3a,b that in the frequency range of 0.1–100 Hz, all samples showed frequency dependence. This showed that the okara beverages fermented with different probiotics all formed unstable, weak gels, and the *G'* of the samples was greater than the *G"*, the elastic components were dominant, and they had solid‐like characteristics (Zhou et al., 2021). The frequency sweep curves of the CK group were higher than those of the other groups, indicating that the strongest gel structure was obtained by non‐fermentation, while the hydrated protein network and gel structure formed after microbial fermentation to produce acid were weaker. The apparent viscosity of all of the samples decreased with increasing shear rate (Figure 3c). Finally, this gradually became smooth and had obvious shear‐thinning characteristics. It is a pseudoplastic fluid with a shear‐thinning phenomenon (Wilbanks et al., 2022). The apparent viscosity difference between all of the samples was not significant (*p* > .05). The Herschel–Bulkley model was used to fit the rheological characteristic curve of the okara beverages, and the results are shown in Table 3. *R* ^2^ represents the fitting accuracy; the higher the value, the better the fitting effect. 0 < *n* < 1 indicates that the okara beverages belonged to the pseudoplastic fluid category.

**TABLE 3:** Rheological parameters of fermented okara beverages with different probiotics.

Sample	Herschel–Bulkley model			
	*τ*_0_ (Pa)	*k* (Pa·s^*n*^)	*n*	*R* ^2^
CK	0.262 ± 0.067^a^	0.613 ± 0.009^a^	0.483 ± 0.003^a^	.9975
Lr	0.009 ± 0.027^b^	0.617 ± 0.014^a^	0.471 ± 0.003^a^	.9975
Lp	0.129 ± 0.018^b^	0.570 ± 0.015^a^	0.483 ± 0.012^a^	.9983
La	0.102 ± 0.115^b^	0.609 ± 0.038^a^	0.477 ± 0.016^a^	.9980
Lr + Lp + La	0.056 ± 0.071^b^	0.604 ± 0.033^a^	0.480 ± 0.013^a^	.9981

*Note*: *τ*_0_ is the yield stress, *k* is the consistency coefficient, *n* is the flow behavior index and *R* ^2^ is the correlation coefficient. Different lowercase letters in the same column a, b indicate significant difference (*p* < .05).

### 3.6 Confocal laser electron microscopy

**FIGURE 4:**
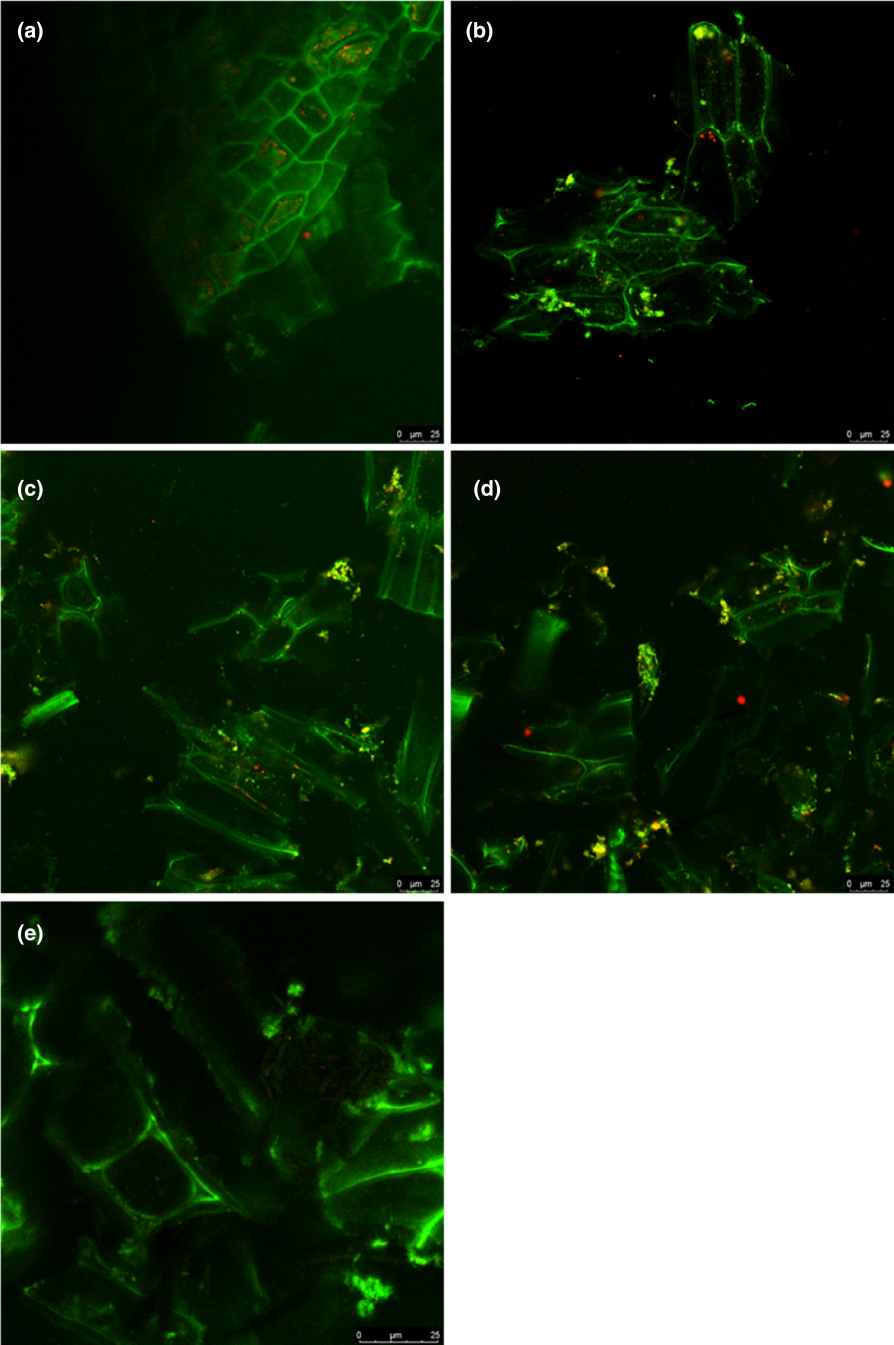
Confocal laser scanning microscopy (CLSM) images of probiotic okara beverages. Protein (green) and fat (red). (A: CK; B: Lr; C: Lp; D: La; E: Lr + Lp + La).

The distribution of protein and fat particles in the okara beverage was observed using CLSM, as shown in Figure 4. The lactic acid produced by probiotic fermentation will lower the pH of the beverage, causing the protein to condense, and proteolytic enzymes will hydrolyze the protein into small pieces, resulting in the precipitation of the protein into smaller pieces. Lipolysis increases the size of the fat globules by destabilizing them. The CK had a dense network structure without obvious faults and fractures. After fermentation, the surface of probiotics was mostly lumpy, and small fragments could be observed. The dense structure inside the bean residue became fluffy and fragile. Castellanos Fuentes et al. (2022) also found similar structures in okara by transmission and confocal microscopy. The results showed that probiotic fermentation could effectively improve the sensory quality of okara and its functional characteristics.

### 3.7 Analysis of the shelf life of probiotic okara beverages

#### 3.7.1 Physicochemical analysis

**FIGURE 5:**
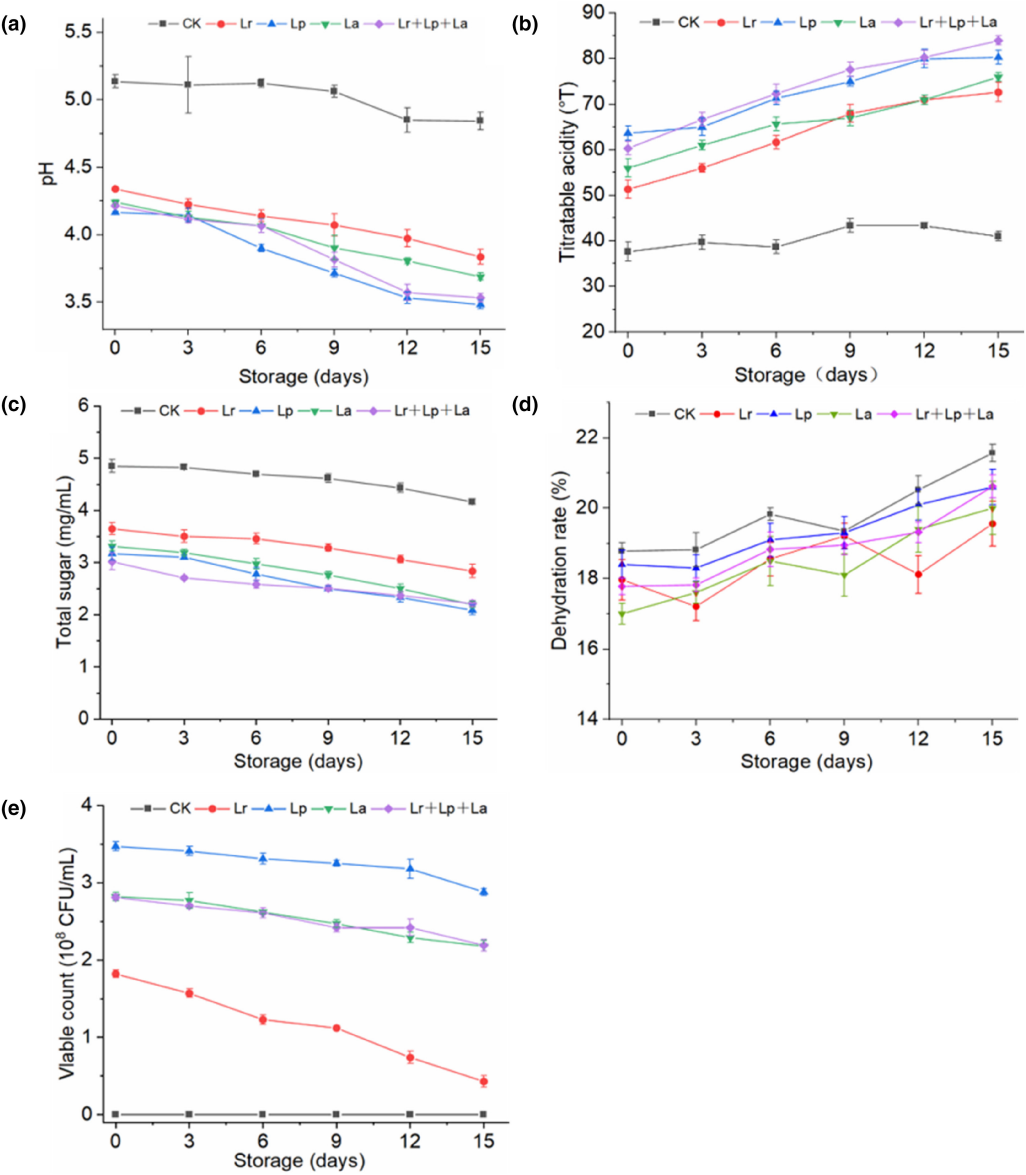
Changes in the physicochemical properties of okara beverages during storage at 4°C. (a) pH; (b) titratable acidity; (c) total sugar; (d) dehydration rate; (e) viable count.

The pH of all of the fermented okara beverages (pH 3.48–3.84) was significantly lower (*p* < .05) compared to the CK group (pH 4.84), while the pH of the CK group changed slightly during storage and remained within the range of pH 4.84–5.14 (Figure 5a). The pH of the Lp group and Lr + Lp + La group showed a maximum rate of decline during the 9–12 days of storage. Low‐temperature storage conditions at 4°C limit the growth and metabolic activity of probiotics. This maintains the taste and texture of the okara‐fermented beverages to a certain extent.

The variation in TAs among the different samples was negatively correlated with the change in pH values (Figure 5b). It can be observed that the acid production of okara beverages fermented with Lp and Lr + Lp + La groups increased from 63.67°T and 60.33°T to 80.33°T and 84°T (*p* < .05), respectively, which were significantly higher than those of the Lr and La groups. In addition, the pH value of the Lr + Lp + La group (pH 3.53) was lower than that of the Lr and La groups (pH 3.84 and 3.69, respectively). Therefore, the lack of acidity of the Lr or La alone was compensated by using mixed strains (Bujna et al., 2018). As storage time increased, some probiotics metabolized to produce acid, resulting in a gradual increase in the total acid content of the affected beverages. Compared with the CK group, the acidity of the 4 okara beverages fermented with probiotics was significantly different during the storage period (*p* < .05). The rapid rise in total acid content after the 6th day may be due to the fact that during storage, the probiotic might have utilized carbohydrates and produced large amounts of organic acid (Zhou et al., 2009).

Carbohydrates were the main nutrients for probiotics, providing them with a rich source of carbon. Compared with the CK group, the total sugar content in 4 probiotic‐fermented okara beverages was significantly reduced (*p* < .05), with a reduction range of 24.74%–37.73% (Figure 5c). Lp group and Lr + Lp + La group had the highest utilization rate of total sugar. During the storage period, the total sugar content of the okara fermented beverages decreased slightly, which was consistent with the results shown by Wang et al. (2019).

As storage time increased, the dehydration rate of the 4 fermented okara beverages also increased (Figure 5d). It is possible that the stability of the network structure formed by the stabilizer (CMC, pectin) and the beverage system decreases. The viscosity of the probiotic beverage gradually decreased, and the centrifugal precipitation rate slightly increased. The CK group was significantly different from the probiotic‐fermented okara beverages (*p* < .05). The centrifugal precipitation rate was the main factor affecting the stability of the beverage during storage. After the storage time was more than 15 days, the appearance of the okara beverage was stratified, and a small amount of supernatant precipitated.

#### 3.7.2 Microbiological viability

To determine the growth abilities of the tested probiotic strains, the growth patterns were monitored during the storage period. The bacterial starter counts in all samples had reached different levels. The Lp group showed the fastest growth after fermentation; the number of viable bacteria in Lp group was 3.53 × 10^8^ CFU/mL, followed by the Lr + Lp + La, La, and Lr groups with 2.77 × 10^8^ CFU/mL and 2.88 × 10^8^ CFU/mL, and 1.77 × 10^8^ CFU/mL, respectively (Figure 5e). All of the samples experienced a gradual decline in the total number of viable probiotic microorganisms as time increased. The Lr group showed the largest decrease, from 1.77 × 10^8^ CFU/mL to 3.47 × 10^7^ CFU/mL. The nutrients in the beverage were continuously consumed, and the growth of probiotics became inhibited. Bifidobacterium is sensitive to pH values below 4.6, and acidification of the okara medium may reduce its viability over time (Saarela et al., 2011). Even the populations in 4 different probiotic‐fermented okara beverages all showed a slight decrease (*p* < .05) within the 15 days. The total viable cell count of probiotics in all fermented samples was consistently above 10^7^ CFU/mL, which can exert its probiotic effect (Mondragón‐Bernal et al., 2017). These results indicate that the okara beverages maintained good health effects during processing and storage.

## 4 CONCLUSION

In conclusion, our results suggest that bioconversion of okara with probiotics may be a method to increase the value of okara. The Lp group has the strongest acid production rate and has great potential for efficient enrichment of bioactive isoflavone glycosides. Due to the different fermentation capabilities of probiotics, they exhibit their own growth and metabolic patterns during fermentation, resulting in different volatile compounds that will lead to differences in flavor characteristics. After fermentation by probiotics, the bad odor of okara beverage was significantly improved; for example, the content of the main bean odor substance [2,4‐decadienal (E, E)‐] was effectively reduced. After fermentation, a large number of esters were produced, resulting in a natural fruit aroma, which had a positive effect on the okara beverage. Most importantly, the okara beverage fermented with probiotics maintained viable counts and had good storage stability during the storage period. This work provides a useful basis for the innovation of probiotic plant‐based beverage production based on okara and fermentation technology and provides a theoretical basis for the flavor improvement of okara products.

## AUTHOR CONTRIBUTIONS

**Yixue Li:** Data curation (equal); formal analysis (equal); investigation (equal); methodology (equal); software (equal); visualization (equal); writing – original draft (equal). **Hong Song:** Conceptualization (equal); funding acquisition (equal); methodology (equal); writing – review and editing (equal). **Zunqin Zhang:** Investigation (equal); methodology (equal). **Ran Li:** Investigation (equal). **Ying Zhang:** Investigation (equal). **Lina Yang:** Validation (equal). **Jun Li:** Supervision (equal). **Danshi Zhu:** Supervision (equal). **Jun Liu:** Visualization (equal). **Hansong Yu:** Visualization (equal). **He Liu:** Funding acquisition (equal); project administration (equal); resources (equal).

## FUNDING INFORMATION

This study was supported by the LiaoNing Revitalization Talents Program (XLYC2002039), National Natural Science Foundation of China (No. 31972031), and National Natural Science Foundation of Liaoning Province (No. 2023‐MS‐293).

## CONFLICT OF INTEREST STATEMENT

The authors declare no conflict of interest.

## ETHICS STATEMENT

This study does not involve any human or animal testing.

## CONSENT FOR PUBLICATION

All the authors are willing for the publication of this manuscript.

## Data Availability

Data will be made available upon reasonable request.
